# Pharmacokinetics, molecular docking, and molecular dynamics simulation unveil novel lichen-derived scaffolds targeting PBP2a MRSA

**DOI:** 10.3389/fbinf.2026.1820903

**Published:** 2026-06-10

**Authors:** Ayushi Priya, N. Venkatesh, Abhishek Rao, Hariom Singh, Sunila Hooda, Lakshay Devrani, Deepansha Raina, Gaurav Selva, Shalini Swami

**Affiliations:** 1 Department of Microbiology, Ram Lal Anand College, South Campus, Benito Juarez Marg, University of Delhi, New Delhi, India; 2 Department of Translational Medicine, AIIMS Bhopal, Bhopal, Madhya Pradesh, India; 3 Department of Biochemistry, Central University of Rajasthan, Ajmer, Rajasthan, India; 4 National Institute of Virology, Indian Council of Medical Research, Maharashtra, India; 5 Department of Biotechnology, Delhi Technological University, Delhi, India

**Keywords:** analogues, docking, methicillin-resistant resistant *Staphylococcus aureus*, methyl orsellinate, simulation

## Abstract

**Introduction:**

Antimicrobial resistance (AMR) is a major threat to global health. It reduces the effectiveness of current antibiotics and treatment for infectious diseases. The rise in AMR is mainly due to the overuse of antibiotics and the increased adaptability of harmful microorganisms. Among resistant bacteria, Methicillin-Resistant *Staphylococcus aureus* (MRSA) can resist an array of antibiotics. A key factor in resistance of MRSA is Penicillin-binding protein 2a (PBP2a). This protein decreases the effectiveness of β-lactam antibiotics and makes treatment more difficult. Therefore, finding new inhibitors that target PBP2a is crucial. In this study*, Parmotrema perlatum*, a himalayan lichen that has not been extensively studied for its antimicrobial properties, was chosen.

**Materials and Methods:**

Phytochemical research identified methyl orsellinate (MO) as a prominent secondary metabolite with antioxidant and antibacterial activities. However, initial docking analysis showed that MO had weak binding affinity for PBP2a. The molecular structure of MO was modified using a scaffold-morphing method to create a series of structural analogues. Molecular docking was conducted to assess their binding affinities and inhibitory potential. A detailed ADMET (Absorption, Distribution, Metabolism, Excretion, Toxicity) screening followed, to evaluate their pharmacokinetic and toxicity profiles. The stability of the top protein-ligand complexes using molecular dynamics (MD) simulations was assessed.

**Results and Discussion:**

MO-1 showed strong binding interactions with PBP2a and maintained stable trajectories throughout the simulation. Furthermore, MM/PBSA analysis indicated negative ΔG values, suggesting favourable binding. Overall, these results indicate that MO-derived analogue, MO-1 could be a computationally prioritised candidate for developing new therapies targeting MRSA. This study aims to open a new avenue to approach the problem of AMR with production of ethno-medicines using MO-1 to create effective therapies against MRSA and help reduce dependency on antibiotics.

## 1 Introduction

**FIGURE 1:**
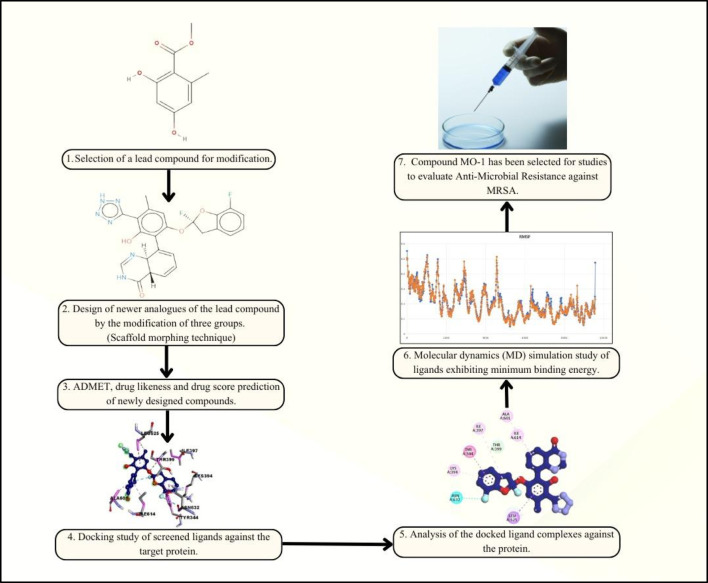
Workflow illustrating the methodology adopted for lead selection, analogue design, absorption, distribution, metabolism, excretion and toxicity (ADMET) prediction, molecular docking, and molecular dynamics (MD) simulation studies against penicillin-binding protein 2a (PBP2a) of methicillin-resistant *Staphylococcus aureus* (MRSA).

Antimicrobial resistance (AMR) is a major global health crisis, with the World Health Organisation attributing ∼1.27 million deaths annually to resistant infections (Halder et al., 2022). This crisis is driven by inappropriate antibiotic use, pathogen adaptability, and increased population mobility, resulting in both community and nosocomial infections. Among resistant bacteria, Methicillin-Resistant *Staphylococcus aureus* (MRSA) represents a critical priority pathogen (Jiao et al., 2023). Its adaptability and resistance mechanisms have challenged therapeutic strategies, necessitating exploration of novel antimicrobial agents and interventions. MRSA expresses PBP2a, encoded by the *mecA* gene, which confers resistance to β-lactam antibiotics (Elabed et al., 2025). PBP2a contains a catalytically active transpeptidase site and a spatially distinct allosteric regulatory site. The fifth-generation cephalosporin, ceftaroline (CFT), exploits this dual-site architecture by inducing allosteric conformational changes that enable active-site inhibition. This dual-site mechanism highlights an exploitable vulnerability for structure-guided inhibitor design targeting both catalytic and regulatory domains of the protein (Masumi et al., 2022). *Parmotrema perlatum*, a himalayan lichen, remains comparatively underexplored for its secondary metabolite diversity (Alqahtani et al., 2021). Phytochemical profiling of *Parmotrema perlatum* identified methyl orsellinate (MO) as a predominant secondary metabolite. MO is a phenolic ester with known antioxidant, anti-inflammatory, and antimicrobial properties (Devi et al., 2021). However, preliminary docking analysis indicated suboptimal binding of MO to PBP2a. To enhance its bioactivity, we employed a scaffold-morphing approach for systematic modification of functional groups to optimise pharmacological performance while maintaining structural integrity (Gupta et al., 2024). Three key positions were targeted based on structure–activity relationship, generating a library of 12 analogues. Comprehensive *in silico* ADMET (Absorption, Distribution, Metabolism, Excretion, Toxicity) screening was conducted to prioritise potential drug-like analogues (Sankaranarayanan et al., 2024). Docking studies were conducted using established computational tools to predict binding affinities and visualise potential inhibitory mechanisms (Masumi et al., 2022). Several analogues demonstrated improved affinity and exhibited strong interactions at both the catalytic and regulatory domains. These findings align with the established allosteric modulation mechanism in PBP2a inhibition (Otero et al., 2013). Molecular dynamics (MD) simulations were then carried out to validate the dynamic stability of the complexes that showed promise of positive results (Halder et al., 2022). This provided insight into the conformational flexibility, the binding stability, and key molecular interaction. Integrating docking with MD simulations enabled assessment of binding stability and conformational adaptability (Fu et al., 2018). Unlike earlier natural-product docking studies, this work integrates scaffold morphing with dual-site PBP2a targeting and long-timescale MD validation. Overall, this study integrates scaffold morphing, dual-site docking, ADMET profiling, and long-timescale MD simulations to prioritise *P*. *perlatum*–derived MO analogues as potential PBP2a inhibitors. Figure 1 represents the procedure followed during the course of the paper.

## 2 Materials and methods

### 2.1 Collection of biological sample

*P. perlatum* (lichen) sample in India was purchased, with the identification confirmed by the herbarium curator. Fresh lichen material was gathered and air-dried before being sent to the laboratory for further studies.

### 2.2 Preparation and extraction of lichen material

140 mL of ethanol was added to 7 g of the dry powdered form of *P. perlatum*, which was then set up in a soxhlet extraction apparatus for 8 h (48–54 cycles). The extract was further evaporated using a vacuum dryer and placed at room temperature until it was completely dry (Alqahtani et al., 2021).

### 2.3 Fourier transform infrared spectrometry and gas chromatography/mass spectrometry analysis

Infrared spectra of the bioactive fraction of the ethanolic extract of *P. perlatum* were recorded on an FT-IR spectrometer (Varian 7000 FT-IR) with a data-processing unit. The small sample under display was ground into a pellet using potassium bromide, and a thin film was prepared by applying pressure. The infrared transmittance spectrum was recorded over a wavenumber range of 400 cm^-1^ to 4,000 cm^-1^. The spectral data were compared with reference data for the identification of the chemical bonds and functional groups existing in the sample (Nautiyal and Dubey, 2021).

GC/MS technique was applied for the detailed identification of the phytocompounds present in the lichen, with 1.5 μL of ethanolic extracts of *P. perlatum*. GC-MS (Shimadzu GCMS-100 QP2010 Plus), was equipped with an AOC-20i + s autosampler and a mass spectrometer. Various conditions were applied for the analysis, such as using the RTX-5 capillary column with helium as the carrier gas, injecting 1.5 μL with a split ratio of 20:1, and a post-column flow rate of 1.21 mL/min. The injection pressure was 85.7 kPa on the column inlet at 250 °C, the ion source temperature was set to 230 °C, and the interface temperature was held at the extensive 260 °C. The oven temperature was set to 80 °C and held for 2 min before ramping to 280 °C by 10 °C/min over 28 min; the total run time was 60 min. The ionization method employed for this was electron impact ionization, and all data were collected by taking full scan mass spectra at a scan speed of 1,250 across the mass-to-charge ratio of 40–600 (Pratibha and Sharma Mahesh, 2016).

### 2.4 Identification of metabolites and lead selection

Out of 20 bioactive compounds obtained through GC/MS analysis, three of them showed significant peaks. For further processing, MO (IUPAC name: Benzoic acid – 2,4-dihydroxy-6-methyl methyl ester) was selected due to its high area percentage, but it showed a weak binding score with PBP2a of MRSA in its native form. MO is a major lichen-derived phenolic ester (42.13% GC–MS peak) which exhibits established activity against Gram-positive cocci, including *Staphylococcus aureus*, and inhibits *Staphylococcus epidermidis* biofilms (∼65%). Its synergism with β-lactams, along with high abundance and favourable ADMET properties, justified scaffold optimization. Hence, a small library of its analogues was prepared through chemical modifications to optimise the structure-activity relationship and enhance both the binding capability with PBP2a as well as the ADMET properties. These obtained bioisosters of MO were then subjected to molecular docking with the target protein (Gupta et al., 2024).

### 2.5 Protein retrieval and selection

The crystal structure of PBP2a from MRSA (PDB ID: 6H5O) was retrieved from the RCSB Protein Data Bank (www.rcsb.org) as a high-resolution X-ray crystallographic structure (2.85 Å resolution) in PDB format. In contrast to the usual closed site in MRSA, PDB ID 6H5O was specifically chosen because of its physiological open PBP2a conformation with piperacillin acylated at SER403, which clearly suggests an allosteric activation that permits inhibitor binding. Furthermore, it offers an ideal active-site geometry for MO-1’s THR399/ILE614 interactions, in contrast to other structures such as 6Q9N. The structure was then visualised using BIOVIA Discovery Studio v25.1.

### 2.6 Characterisation of allosteric and active sites

PBP2a catalyses dd-transpeptidation during MRSA cell wall biosynthesis, conferring β-lactam resistance. It features catalytic SER403 (SXXK motif) protected by dynamic α2-α3 and R loops within its C-terminal transpeptidase domain. Site characterisation integrated crystallographic (PDB: 6H5O, 3ZG5, 5M18) and kinetic studies with BIOVIA Discovery Studio v25.1 modelling.

The allosteric site (∼residues 240–350, 60 Å from active site) contains hydrophobic (TYR105, PHE276, ILE242) and polar residues (ASN104, ASN146). CFT binding induces α2-α3 loop shifts, opening the occluded active site centered on SER403, with oxyanion hole (ASN464, LYS468), acyl acceptor (THR599, SER462), and positioning residues (GLU447, TYR446) (Chiang et al., 2020). Discovery Studio mapped side-chain properties and interactions (H-bonds, π-π stacking), guiding MO analogue design for dual-site targeting (Rahmadani et al., 2025).

### 2.7 Ceftaroline as foundational reference for dual-site docking

**FIGURE 2:**
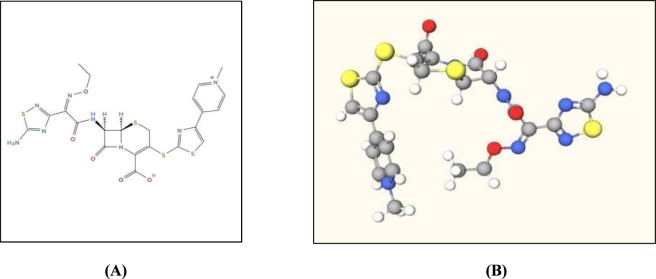
**(A)** Two-dimensional (2D) and **(B)** three-dimensional (3D) chemical structure of ceftaroline (CFT), the reference anti-MRSA cephalosporin used as the standard ligand for docking studies against PBP2a.

CFT, the only FDA-approved cephalosporin effective against MRSA PBP2a, served as the gold-standard reference for our docking studies due to its unique allosteric modulation mechanism (Figure 2) (Jiao et al., 2023). Unlike conventional β-lactams rejected by PBP2a’s occluded active site, CFT employs a two-step binding strategy: first occupying the allosteric site (∼60 Å from SER403), inducing conformational opening of protective loops (α2-α3, R-loop), then accessing the active site for covalent inhibition. CFT binding at the allosteric pocket (TYR105, ASN104, ASN146, PHE276) triggers signal propagation through residue networks, destabilising the SER403 active site closure (Chiang et al., 2020). This 60 Å allosteric pathway repositions loops, exposing the catalytic serine for secondary CFT acylation. Crystal structure PDB: 6H5O, which is piperacillin bound at the allosteric site, confirm this open conformation, validating CFT’s efficacy against otherwise resistant MRSA. In contrast to piperacillin, which shows β-lactamase synergy with tazobactam rather than direct inhibition, CFT inhibits PBP2a by specifically binding at an allosteric site to open the occluded pocket. However, its limitations include emerging resistance via allosteric site mutations (N146K), intravenous-only administration restricting outpatient use, limited oral bioavailability, and high production costs associated with its complex cephalosporin scaffold (Otero et al., 2013; Jiao et al., 2023). These shortcomings underscore the therapeutic need for novel chemical scaffolds that retain CFT’s allosteric activation capability while offering improved pharmacokinetic properties and resistance profiles. Our MO analogues were designed to mimic CFT’s allosteric activation while potentially offering improved physicochemical properties. The superior docking scores and MD stability of MO analogues position them as computationally superior candidates for further development.

### 2.8 Design of bioisosteric analogues of methyl orsellinate

**FIGURE 3:**
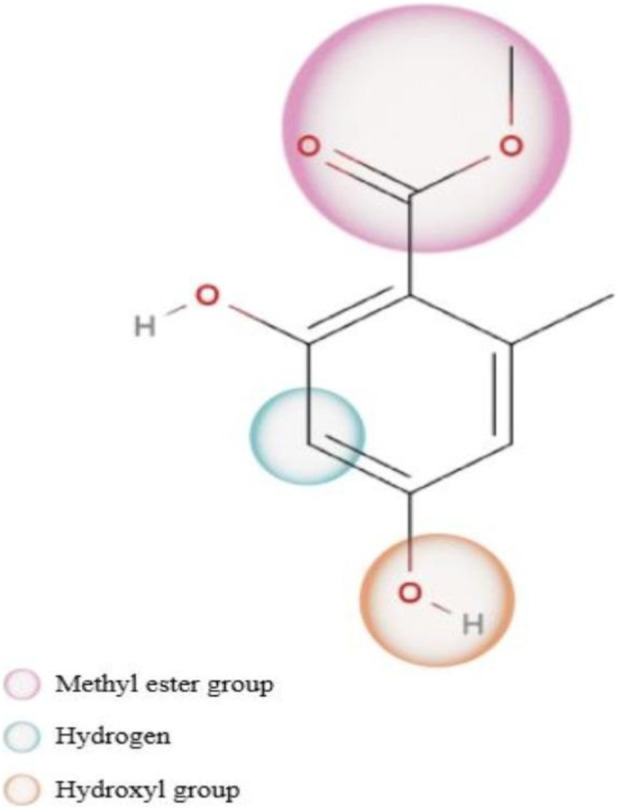
Chemical structure of methyl orsellinate (MO) and the bioisosteric modifications introduced for analogue design. Functional group substitutions were incorporated to improve molecular interactions, pharmacokinetic properties, and binding affinity toward PBP2a.

MO was identified as the lead compound in preliminary analysis and was used as the basis for designing a small library of analogues. Bioisosterism refers to a drug design technique in medicinal chemistry in which an atom/group of atoms is replaced by a different functional group possessing specific physical and chemical properties, aiding in achieving similar biological results (Khattab and Al-Karmalawy, 2021; Thakur et al., 2025). Bioisosteric substitutions and scaffold morphing were employed to generate analogues of MO. Three substitution sites on the MO scaffold (1′, 3′, and 4′) were selected based on structural analysis (Figure 3). At these positions, diverse bioisosteric groups—tetrazole, imidazole derivatives, pyrazole, tetrahydrofuran, amides, pyridine, and fluorinated substituents were added to increase its antibacterial property, as observed in the results of FTIR. Based on these bioisosteric substitutions, a library of twelve analogues (MO-1 to MO-12) was created using MolView (https://molview.org/) for further analysis.

### 2.9 Pharmacokinetic and toxicity screening (ADMET analysis) pKCSM and SwissADME

ADMET properties were predicted using SwissADME (Fernando et al., 2019) and pkCSM (Pires et al., 2015) *in silico* tools. Canonical SMILES (Simplified Molecular Input Line-Entry System) of all compounds were used as input for both servers. The resulting output is based on several parameters that determine the drug likeness of a compound. These parameters assess drug permeability (CaCO_2_, intestinal absorption, CNS and BBB), systemic clearance via hepatic or renal pathways, and safety profiles including hepatotoxicity, AMES toxicity, skin sensitisation, and maximum tolerated dose in humans. Drug-likeness was studied using Lipinski’s and Veber’s criteria (Pires et al., 2015).

### 2.10 Molecular docking

#### 2.10.1 Protein preparation

The PBP2a structure was obtained from the PDB (PDB ID: 6H5O) in. pdb format. The protein was then prepared for docking analysis by removing crystallographic water molecules and other unnecessary heteroatoms, as they would interfere with the specific interactions between the targeted drug and protein, and would unnecessarily increase the grid box size. Kollman charges were then assigned to the relevant regions. Finally, energy minimisation was performed using Discovery Studio (McGreig et al., 2022).

#### 2.10.2 Ligand preparation

The twelve analogues (MO-1 to MO-12) were converted from their SMILES notations into 3D PDB structures through Open Babel 2.4.1. All analogues were geometry-optimised and energy-minimised before molecular docking to ensure conformational stability and realistic binding poses (Azam and Abbasi, 2013; Otero et al., 2013).

#### 2.10.3 Molecular docking studies

Docking analysis was performed using AutoDock Vina 1.1.2. The active and allosteric sites targeted for docking were based on previous studies showing CFT-induced conformational changes, and our designed structures were docked to evaluate analogous interactions (Masumi et al., 2022; Elabed et al., 2025; Rahmadani et al., 2025). Two grid boxes were designed for each binding site to facilitate site-specific docking. The dimensions of the grid for the allosteric site were fixed at X = 58, Y = 62, Z = 66 and centred at X = −6.778, Y = 0.265, Z = 71.228. Likewise, the gird dimensions for the active site were optimised at X = 52, Y = 46, Z = 52 and centred at X = 29.667, Y = −6.257, Z = 25.72. Vina software was run in energy range = 4 and exhaustiveness = 8. The docking results were visualised using PyMOL 3.1.6.1 and Discovery Studio Visualizer v25.1 (Pieroni et al., 2023).

### 2.11 Analysis of protein-ligand interaction

Protein-ligand interaction studies were analysed by identification of hydrogen bonds, alkyl interactions, halogen bonds, and π-σ interactions, for both the active site and allosteric site. Binding affinity and specificity were further evaluated using MM/GBSA scores using Schrödinger-Prime (Mirzadeh et al., 2022; Sankaranarayanan et al., 2024).

### 2.12 Molecular dynamics simulations

MD simulations were conducted to examine the temporal stability and interaction behaviour of selected complexes using the CHARMM36 all-atom force field. Systems were solvated in explicit TIP3P water within cubic boxes (∼10 Å solute-buffer distance) under periodic boundary conditions in all dimensions. Simulations utilised the NPT ensemble at physiological conditions: 310 K (Nose-Hoover thermostat, τ = 0.1 ps) and 1 atm (Parrinello-Rahman barostat) (Sankaranarayanan et al., 2024). The Leapfrog integrator was applied with a 2 fs timestep. Electrostatic interactions were treated using the Particle Mesh Ewald (PME) method (1.0 nm cutoff, 0.16 nm Fourier spacing), with Van der Waals interactions using a 1.0 nm potential-shifted Verlet cutoff. Each complex underwent 100 ns production runs (50 million steps), preceded by energy minimisation, NVT, and NPT equilibration. Trajectories were analysed for structural stability (RMSD, RMSF, radius of gyration), hydrogen bonding, and MM/PBSA binding free energies using Schrodinger-2024 Desmond software tools on high-performance computing clusters (Thakur et al., 2025).

## 3 Results

### 3.1 FT-IR and GC/MS analysis

FTIR analysis has revealed a distinctive band absorption peak at 3,235.80 cm^-1^, indicating the free hydroxyl phenolic group (OH) stretching (Supplementary Figure S1 in Supplementary Material). This strongly indicates the presence of phenolic compounds, known for their antioxidant activity. Peaks at 2,919.16 cm^-1^ and 2,849.1 cm^-1^ indicate C-H stretching and bending, such as in alkanes (Supplementary Figure S2 of Supplementary Material). The band absorption peak at 1,646.94 cm^-1^ indicates a C=O stretching aromatic ring or aliphatic chain. Band peaks at 1,000, 1,062, 1,106, 1,163, 1,201.7, 1,263.8, and 1,317.13 cm^-1^ are due to the vibration of the (C-O) polyol stretching group, such as hydroxyl flavonoids, indicating the abundance of phenolic and flavonoid compounds, which could be the key components responsible for antimicrobial activity against ESKAPE pathogens (Supplementary Figure S3; Supplementary Table S1 of Supplementary Material). The additional bioactive compounds at low concentrations with their retention time, percentage area (concentration), and molecular formula are provided in the Supplementary Material.

### 3.2 Structural insights into PBP2a binding sites

PBP2a exhibits a dual-site architecture comprising an occluded active site and a conformationally coupled allosteric site. The active site revolves around the Ser403-Ser462 diacylglyceride nucleophile, supported by TYR446, GLN438, ASN464, THR600, and HIS597. In the apo form, TYR446 stacks parallel to the SER462 hydroxyl, while HIS597 coordinates via Nε···Oγ H-bond (2.8 Å), and GLN438-ASN464 form a polar cluster that rigidifies the β3–β4 loop. THR600 anchors the SxN motif, limiting cleft access and thwarting acylation—key to hydrolysing β-lactams before covalent reaction. The allosteric site, ∼25 Å distant, encompasses LYS76, ASN104, TYR105, ILE144, ASN146, TRP205, ASN204, GLY271, LYS273, TYR272, GLU294, and TRY297. Polar anchors include ASN104 (amide donor to ligands), ASN146 (side-chain H-bond acceptor), and charged LYS273-GLU294 pairs modulating loop dynamics. The GLY271 hinge enables flexing of the Ω-loop (residues 270–280), coupling to helix α2–α3 (144–205). CFT exemplifies allosteric modulation: its cephalosporin core H-bonds ASN104 (2.9 Å) and ASN146, with the aminothiazole π-stacking TYR105 (4.0 Å). This displaces the α3 helix, rotating TYR105 to weaken active-site TYR446 packing and dilate the cleft by 3–5 Å. MD studies reveal propagated effects: RMSF increases in GLY271 (1.5 Å) loosen the Ω-loop, exposing SER462. Crystal structures (PDB: 4CJM) confirm CFT’s low-micromolar affinity stems from this indirect activation, bypassing direct active-site occlusion.

These sites represent exploitable vulnerabilities: allosteric priming enhances β-lactam access, while direct active-site ligands could synergize for potent inhibition. Future designs targeting residue clusters (e.g., ASN104-TYR105, SER403-GLN438) hold promise against resistant isoforms.

### 3.3 Bioisosteric modifications and structural analysis

**FIGURE 4:**
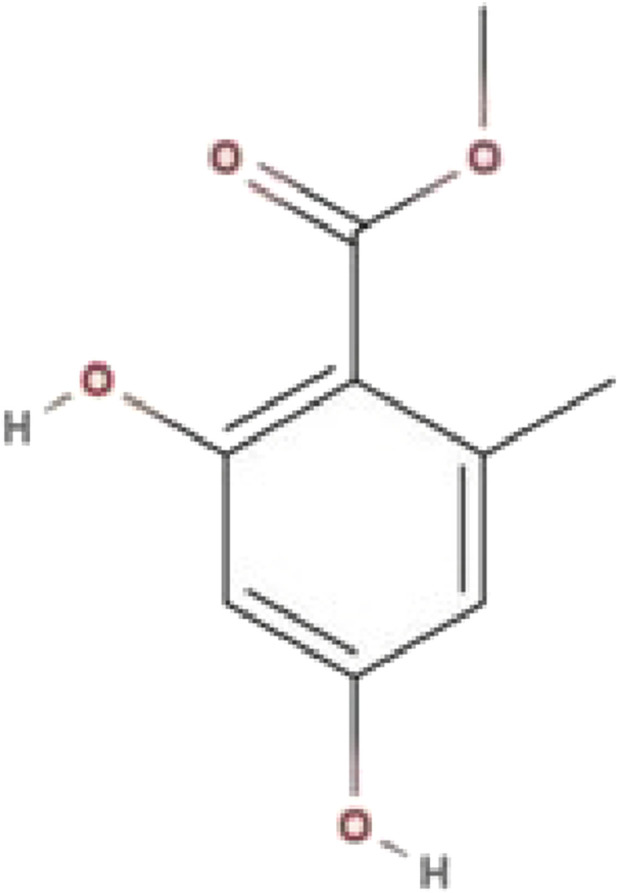
Two-dimensional (2D) chemical structure of methyl orsellinate (MO), the parent molecule used for the design of bioisosterically modified analogues.

Schematic 2D diagram of MO is shown below (Figure 4). Structurally distinct analogues were designed by introducing varied heterocyclic rings. These modifications were introduced to alter electronic distribution, hydrogen bonding, and steric bulk, optimising binding with PBP2a sites (Table 1). The combined electronic, steric, and biological properties support both enhanced target affinity and antibacterial efficacy.

**TABLE 1:** Bioisosterically designed analogues of methyl orsellinate (MO) showing the functional group modifications introduced to improve binding affinity, pharmacokinetic properties, and anti-MRSA potential against penicillin-binding protein 2a (PBP2a).

Analogue	Position at which changes are made	Original group present in MO	The group that is added or replaced in the parent MO	Structure
MO-1	1/3/4	-COOCH_3_/-H/-OH	Tetrazole derivative/imidazopyridine polycyclic ring/dioxolane fluorophenyl ring	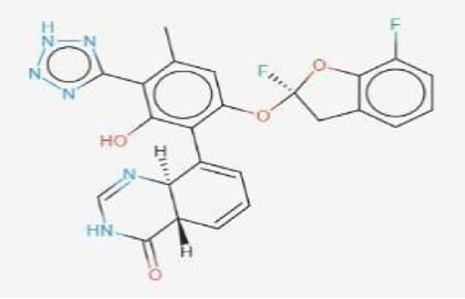
MO-2	1/3/4	-COOCH_3_/-H/-OH	Tetrazole derivative/indole-like bicyclic fused system/fluorobenzoyl group	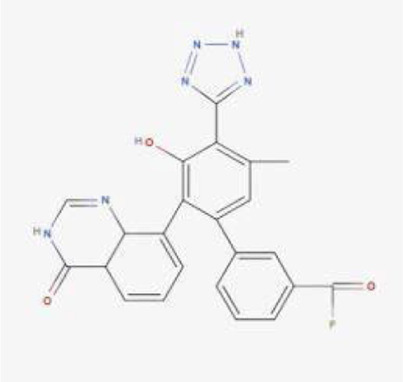
MO-3	1/3/4	-COOCH_3_/-H/-OH	Tetrazole derivative/fused pyrazole-pyrazine/2-fluoro-4-aminophenyl-morpholine derivative	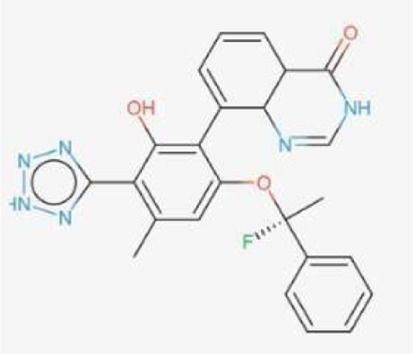
MO-4	1/3/4	-COOCH_3_/-H/-OH	Tetrazole derivative/pyrazolo[4,3-d] pyrimidin-4-one core/3,3-difluorooxolane ring	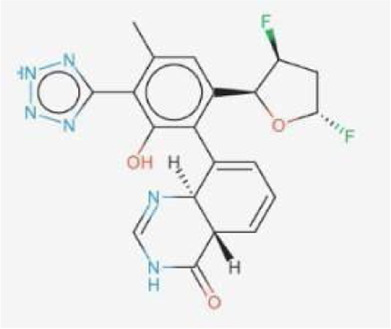
MO-5	1/3/4	-COOCH_3_/-H/-OH	Tetrazole derivative/fused pyrazole-pyrazine/3-(trifluoromethoxy)benz ene	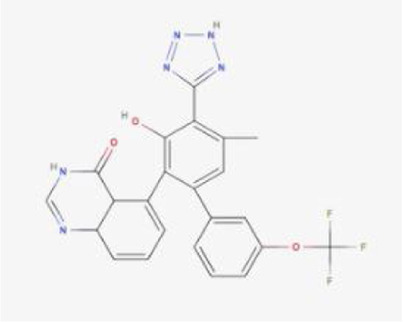
MO-6	1/3/4	-COOCH_3_/-H/-OH	Tetrazole derivative/fused pyrazole-pyrazine/2-fluoro-4-methylquinoline	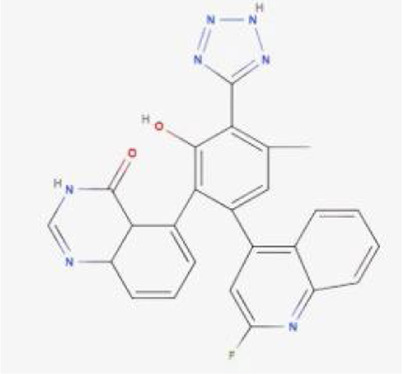
MO-7	1/3/4	-COOCH_3_/-H/-OH	Tetrazole derivative/pyrazolo-pyrimidinone/morpholine derivative/3-fluoro-5-methyl-oxane	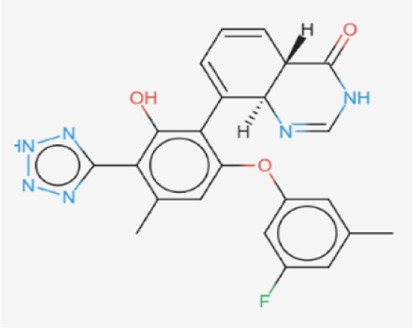
MO-8	1/3/4	-COOCH_3_/-H/-OH	Tetrazole ring/Pyrazolo-pyrimidine fused derivative/difluoro-dioxolane	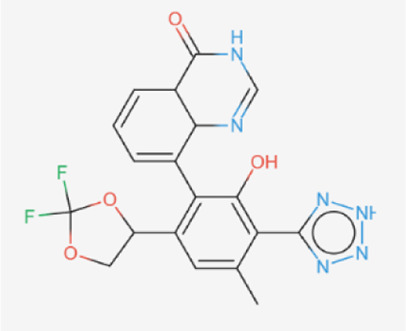
MO-9	1/3/4	-COOCH_3_/-H/-OH	Tetrazole ring/pyrazolo-pyrimidine/tetrafluorooxetane	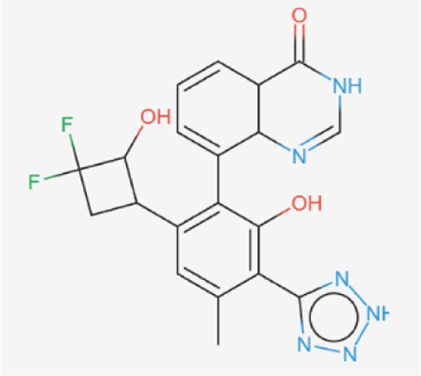
MO-10	1/3/4	-COOCH_3_/-H/-OH	Tetrazole structure/pyrazolo-pyrimidine derivative/fluoro-dioxolanyloxy benzene derivative	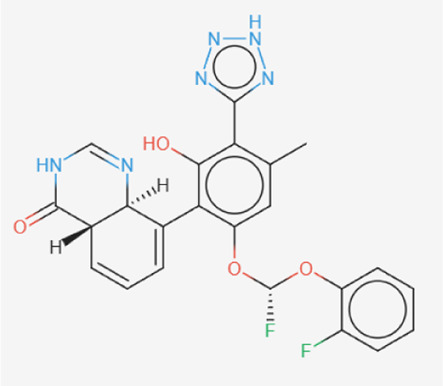
MO-11	1/3/4	-COOCH_3_/-H/-OH	Tetrazole derivative/pyrazolo-pyrimidine derivative/2-fluoro-phenolic derivative	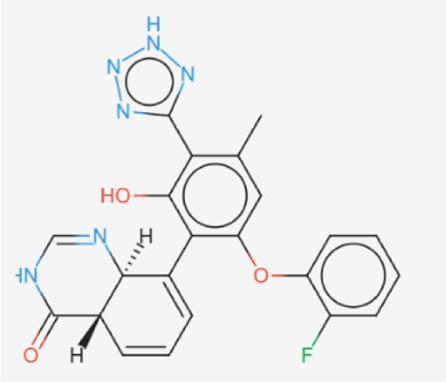
MO-12	1/3/4	-COOCH_3_/-H/-OH	Tetrazole derivative/pyrazolo-pyrimidine derivative/fluorophenylmethanol	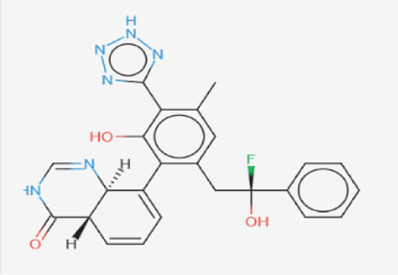

### 3.4 In silico pharmacokinetic study (ADMET analysis)

Predicted ADMET profiles were used to prioritise analogues with favourable pharmacokinetic properties. ADME properties determine the efficacy and safety of the desired compound within the body, highlighting its ability to reach the target site, remain sufficiently abundant for therapeutic activity, and undergo efficient clearance (Table 2). Optimal ADME characteristics ensure high bioavailability, potency, and safety, allowing for efficient absorption, targeted delivery, controlled metabolism and timely elimination from the body to prevent any side effects and achieve clinical success. In our study, we used two tools, SwissADME and pkCSM to evaluate the ADME properties of the 12 bioisosters. The predictable CaCO2 permeability scores and intestinal absorption values provide valuable insights into a substance’s ability to access intestinal cell membranes, a critical aspect of oral drug absorption. The CaCO2 scores and absorption values for all 12 analogues show high predicted permeability, indicating high oral bioavailability, which is crucial for therapeutic drugs. MO-8 represents the highest CaCO2 value of 0.964, and MO-2 has the lowest score of - 0.237, while MO-5 shows the highest value for intestinal absorption value and MO-9 is the least.

**TABLE 2:** Predicted absorption, distribution, metabolism, excretion and toxicity (ADMET) properties of methyl orsellinate (MO) analogues calculated using pkCSM and SwissADME computational tools.

Analogue	Water solubility (log mol/L)	CACO2 Permeability (LogPapp in 10^−6^ cm/s)	Intestinal absorption (%)	PGP Substrate	BBB Permeability (LogBB)	CYP2D6 Inhibitor	Total clearance (logmL/min/kg)	Renal OCT2 Substrate	CNS Permeability (log cm/s)	AMES Toxicity	Max tolerated dose (mg/kg)	Hepatotoxicity	Skin sensitization	Minnow toxicity (log mg/L)
MO-1	−3.391	0.499	76.225	Yes	−1.726	No	−0.307	No	−4.223	Yes	0.608	Yes	No	1.121
MO-2	−3.193	−0.237	61.733	Yes	−1.659	No	0.715	No	−3.94	No	0.636	Yes	No	2.076
MO-3	−3.044	−0.047	69.972	Yes	−1.379	No	0.736	No	−4.171	No	0.337	Yes	No	2.673
MO-4	−2.744	0.319	74.546	Yes	−1.607	No	−0.287	No	−4.471	No	0.190	Yes	No	2.655
MO-5	−3.2	−0.182	69.29	Yes	−1.724	No	0.716	No	−3.865	No	0.654	Yes	No	1.83
MO-6	−3.139	−0.147	82.451	Yes	−1.655	No	−0.037	No	−3.82	No	0.607	Yes	No	1.564
MO-7	−3.576	0.192	76.001	Yes	−1.605	No	−0.146	No	−3.605	Yes	0.821	Yes	No	0.84
MO-8	−2.939	0.964	66.259	Yes	−2.031	No	0.531	No	−3.984	No	0.458	Yes	No	3.158
MO-9	−2.946	0.918	58.679	Yes	−1.779	No	0.539	No	−3.89	No	0.521	Yes	No	2.881
MO-10	−3.538	0.102	72.381	Yes	−1.821	No	−0.303	No	−4.31	No	0.543	Yes	No	1.35
MO-11	−3.287	0.173	78.779	Yes	−1.503	No	−0.24	No	−3.9	No	0.715	Yes	No	1.769
MO-12	−3.473	0.747	66.619	Yes	−1.465	No	0.002	No	−4.056	No	0.822	Yes	No	1.884

Total clearance is another important parameter to determine how quickly and efficiently a drug is removed from systemic circulation. Drugs with moderate total clearance are preferred because they avoid rapid elimination but do not accumulate as well. For most drug-like molecules, log clearance values between −0.5 and +1 are considered reasonable for therapeutic applications. The log clearance values for majority of the compounds lie near or around the ideal range, with MO-3 having the most ideal value of 0.736. MO-1 has the lowest score of −0.307 while still being in an acceptable range. AMES toxicity is another critical parameter to determine the potential of a compound to cause mutations in DNA, as measured by the AMES test. Most of our analogues do not exhibit AMES toxicity, indicating no mutagenic activities and are safe for therapeutic purposes.

Now, MO-1 demonstrates a well-balanced ADMET profile, thus favourable for further investigation (Table 2). Its CaCO2 permeability value of 0.499 and intestinal absorption of 76.2% indicate highly efficient transport across the intestinal epithelium. Its blood-brain barrier permeability and CNS permeability values are quite low, suggesting minimal CNS exposure and reducing the likelihood of CNS-related side effects. Its total clearance rate also suggests it would be eliminated at a manageable pace, thereby minimising the risk of accumulation. However, its AMES toxicity was positive, indicating some mutagenic concerns. MO-1 remains the lead compound due to its strong MD stability, favourable MM/GBSA binding, and high PBP2a affinity (−9.8/−8.4 kcal/mol). Toxicity risks can be mitigated through microdosing, targeted structural modifications (tetrazole, acylhydrazone, fluorine repositioning, N-methylation), and validation via bacterial mutation and MRSA MIC assays.

MO-2 also displays computationally superior pharmacokinetic attributes (Table 2). Its CaCO2 permeability score of −0.237 and intestinal absorption estimate of 61.7% represent good and reliable systemic uptake through the gut. MO-2 also shows limited CNS penetration and hence low probability of undesirable CNS effects, and a total clearance value (0.715) points to efficient elimination, indicating safe plasma concentrations during therapy. The compound is also negative for AMES toxicity, which is favourable and highlights its safety potential as a clinical drug.

### 3.5 Comparative analysis of pkCSM and SwissADME results

The comparative analysis utilising both pkCSM and SwissADME provides a strong correspondence in determining essential pharmacokinetic and ADME properties, supported by both Cohen’s kappa analysis and percentage agreement (Table 3). For crucial parameters like blood-brain barrier (BBB) permeability and inhibition of CYP450 enzymes, both the kappa values and percentage agreement show perfect results of 1.0% and 100%, respectively, indicating that the predictions made by both tools are identical. Intestinal adsorption and PGP substrate status show moderate agreement. The percentage agreement values are 83.33%, and the kappa values are around 0.6, highlighting significant but not absolute consistency (Table 4). For parameters like water solubility and CYP1A2 inhibition, the percentage agreement values of 75% and 66.67% and kappa values in the range 0.3–0.35, demonstrate increased variability and inconsistencies between the predictions. These results highlight that while pkCSM and SwissADME are highly reliable for predicting pharmacokinetic parameters, some parameters show variable results and hence employing both the tools in conjunction can enhance the confidence and reliability of ADME profiling, especially in drug discovery.

**TABLE 3:** Percentage agreement between pkCSM and SwissADME predictions for selected pharmacokinetic parameters like absorption, distribution, metabolism, excretion and toxicity (ADMET) of methyl orsellinate analogues.

Parameter	Percentage agreement
Intestinal absorption	83.33%
BBB permeability	100.00%
Water solubility	75.00%
PGP substrate	83.33%
CYP1A2 inhibitor	66.67%
CYP2C19 inhibitor	100.00%
CYP2C9 inhibitor	100.00%
CYP2D6 inhibitor	100.00%
CYP3A4 inhibitor	100.00%

**TABLE 4:** Interpretation scale for Cohen’s kappa coefficient values at 95% confidence interval used to evaluate the level of agreement between pkCSM and SwissADME predictions.

Parameter	Kappa value	95% CI lower	95% CI upper	Kappa range	Interpretation
Intestinal absorption	0.605	0.0	1.0	0.60–0.79	Moderate agreement
BBB permeability	NaN	NaN	NaN	1.0	Perfect agreement (all identical predictions)
Water solubility	0.356	−0.25	1.0	0.21–0.40	Fair agreement
PGP substrate	0.582	−0.001	1.0	0.40–0.59	Moderate agreement
CYP1A2 inhibitor	0.317	0.0	0.8	0.21–0.40	Fair agreement
CYP2C19 inhibitor	NaN	NaN	NaN	1.0	Perfect agreement (all identical predictions)
CYP2C9 inhibitor	NaN	NaN	NaN	1.0	Perfect agreement (all identical predictions)
CYP2D6 inhibitor	NaN	NaN	NaN	1.0	Perfect agreement (all identical predictions)
CYP3A4 inhibitor	NaN	NaN	NaN	1.0	Perfect agreement (all identical predictions)

NaN, not a number NaN values for confidence intervals indicate perfect agreement without a computable CI.

**FIGURE 5:**
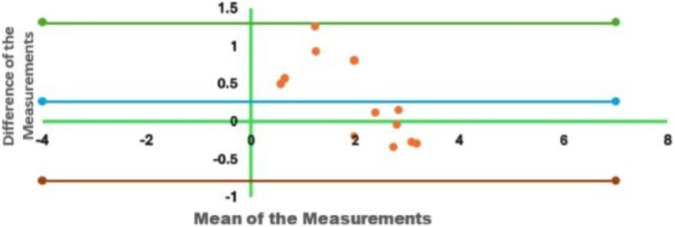
Bland–Altman plots showing the agreement between predicted logarithm of partition coefficient (logP) values obtained from pkCSM and SwissADME tools for the designed methyl orsellinate analogues. The central line represents the mean difference, while the upper and lower lines indicate the 95% limits of agreement.

The Bland–Altman plot is another graphical method used to analyse the agreement between two quantitative measurements by plotting the difference between paired observations against their mean (Figure 5). It was also employed to evaluate the agreement between ADME property predictions from pkCSM and SwissADME, which supported the findings, by showing a mean bias of 0.267 ± 0.54, with 95% limits of agreement (LOA) ranging from −0.784 to 1.31. Importantly, 100% of the data points fall within the 95% LOA, indicating a good level of agreement between the two tools for logP prediction.

### 3.6 Molecular docking

To validate the docking method, a self-docking technique was performed (Supplementary Figures S5, S6 of Supplementary Material). The co-crystallised ligand was extracted from PDB, redocked into the native site using the same settings, and then superimposed in PyMol 3.6.1. The superimposed residues were then highlighted using LigPlot v.2.3.1 software. With a binding affinity of −6.1 kcal/mol, it yielded an RMSD of 0.00 Å, indicating nearly identical alignment. TRY519, GLU602, SER403, GLY599, and ALA601 were important residues in the self-docking interaction.

**FIGURE 6:**
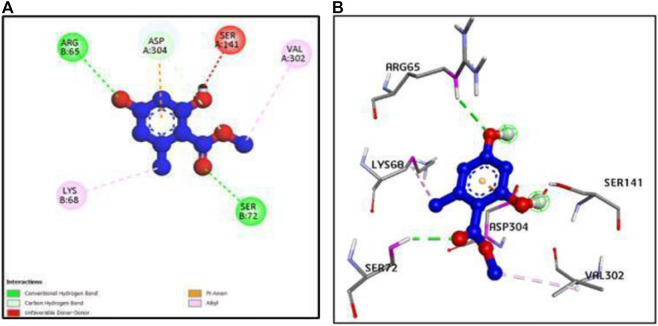
**(A)** Two-dimensional (2D) and three-dimensional (3D) interaction poses of methyl orsellinate (MO) docked at the allosteric site of penicillin-binding protein 2a (PBP2a) from methicillin-resistant *Staphylococcus aureus* (MRSA). **(B)** Two-dimensional (2D) and three-dimensional (3D) interaction poses of methyl orsellinate (MO) docked at the active site of PBP2a.

**FIGURE 7:**
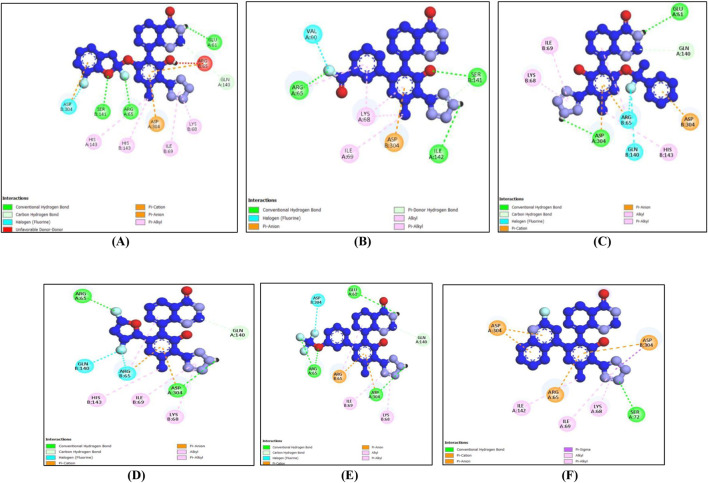
Two-dimensional (2D) interaction diagrams of methyl orsellinate analogues (MO-1 to MO-6) docked at the allosteric site of penicillin-binding protein 2a (PBP2a) from methicillin-resistant *Staphylococcus aureus* (MRSA), showing hydrogen bonding, hydrophobic interactions, and π-interactions with amino acid residues. **(A)** MO-1 **(B)** MO-2 **(C)** MO-3. **(D)** MO-4 **(E)** MO-5 **(F)** MO-6.

For the parent molecule (MO), the docking scores of −5.5 and −4.6 were obtained at the allosteric and active site respectively (Figure 6). Docking with CFT at the allosteric site showed a binding score of −7.2 and −6.4 at the active site. Subsequently, the docking with analogues produced a better score, highlighting increased binding affinity as compared to the parent molecule. At the allosteric site, MO-1 demonstrated a binding score of −9.8 kcal/mol. In the ADV docked complex, both LYS B68 and ILE B69 interact with the tetrazole ring via a π-alkyl interaction. HIS A143 and HIS B143 forms π-alkyl interaction with the methyl group of the MO ring and the fluorinated benzene ring, respectively. SER B141 and ARG A65 form a hydrogen bond with the oxygen atom and the fluorine atom of the THF ring, respectively, while ASP B304 interacts with fluorinated benzene via two interactions: a π-cation with the benzene ring and a halogen bond with the fluorine atom (Figure 7). ASP A304 interacts with the MO ring via π-anion interaction, and ARG B65 forms a π-cation bond with the MO ring and an unfavourable donor-donor interaction with the H atom of the hydroxyl group of MO. GLU A61 forms a hydrogen bond with the NH group of the β-lactam ring, and lastly GLN A140 forms a carbon-hydrogen bond with the lactam ring (Tables 5, 6). MO-1 exhibited the most-favourable binding score of −8.4 kcal/mol at the active site. In the ADV docked complex, the ALA A601 and ILE A614 forms alkyl and pi-alkyl interactions with the indole ring of the analogue, stabilising the hydrophobic core. Additionally, ILE A397, LYS A394 form similar alkyl interactions with the tetrahydrofuran ring and fluorinated benzene ring, respectively, while THR A399 forms a hydrogen bond with the ether oxygen. TYR A344 interacts with the fluorinated benzene ring via a π-π T-shaped bond, and LEU A525 forms 2 bonds with the MO ring, a π sigma bond and an alkyl interaction with the methyl group attached to the C6 position. Finally, ASN A632 forms a halogen bond with the fluorine atom of the benzene ring (Figure 9).

**TABLE 5:** Molecular docking scores and binding affinities of methyl orsellinate analogues at the allosteric site of penicillin-binding protein 2a (PBP2a) from methicillin-resistant *Staphylococcus aureus* (MRSA).

Analogue	Docking score	MM/GBSA Score	Hydrogen bonds	Other interactions
MO-1	−9.8	−46.48	SER B141, ARG A65, GLU A61	ASP B304, ASP A304, HIS A143, HIS B143 ILE B69, LYS B68, GLN A140, ARG B65
MO-2	−9.1	−50.84	ARG A65, ILE A142, SER B141	VAL A60, LYS A68, ILE A69, ASP B304
MO-3	−9	−38.46	GLU A61, ASP A304	GLN A140, HIS B143, ARG B65, GLN B140, LYS B68, ILE B69
MO-4	−8.7	−46.32	ARG A65, ASP A304	GLN B140, LYS B68, ILE B69, HIS B143, ARG B65
MO-5	−8.9	−43.44	GLU A61, ARG A65, ASP A304	LYS B68, ILE B69, GLN A140, ARG B65, ASP B304
MO-6	−9.1	−48.11	SER A72	ASP A304, ASP B304, ILE A142, LYS AA68, ILE A69, ARG A65
MO-7	−8.3	−39.72	SER B72	LYS B68, GLN A140, ASP B139, ASP A304, ASP B304 ARG B65
MO-8	−8.6	−37.00	ASP A304	GLN A140, ARG B65, LYS B68, ILE B69, ASP B304
MO-9	−8.5	−41.87	ASP A304	ASP a 139, GLN A140, LYS B68, ILE B69, HIS B143, ARG B65
MO-10	−9.2	−55.82	ASP A304	LYS B68, ILE B69, ARG A65, ASP B304, LYS A68, ASP A139, GLN A140, ARG B65
MO-11	−8.5	−48.61	ASP A304	ARG B65, GLN A140, LYS B68,
MO-12	−8	−45.46	GLU A170	PRO A358, MET A372, VAL A256, VAL A277, ARG A241

**TABLE 6:** Molecular docking scores and binding affinities of methyl orsellinate analogues at the active site of penicillin-binding protein 2a (PBP2a) from methicillin-resistant *Staphylococcus aureus* (MRSA).

Analogue	Docking score	MM/GBSA Score	Hydrogen bonds	Other interactions
MO-1	−8.4	−46.48	THR A399	ILE A614, ALA A601, ILE A397, TYR A344, LYS A394, ASN A632, LEU A525
MO-2	−8.1	−50.84	LYS A281	LYS A285, LEU A286, PRO A497, LEU A391
MO-3	−7.6	−38.46	THR A399, LYS A634	ILE A614, TYR A344, LEU A525
MO-4	−7.5	−46.32	GLY A282, TYR A366	PRO A497, TYR A496, HIS A251, LYS A281, LEU A391
MO-5	−7.4	−43.44	LYS A281, TYR A499, GLN A396	LEU A286, PRO A497, TYR A366, LEU A391
MO-6	−7.4	−48.11	ASN A464, THR A600, LYS A597,	SER A403, ALA A642, MET A641, GLU A602
MO-7	−7.4	−39.72	LYS A634, THR A398, SER A400	ALA 601, THR A399, ILE A614, TYR A344
MO-8	−7.3	−37.00	LYS A281	LEU A391, GLY A282
MO-9	−7.3	−41.87	LYS A281, TYR A496, HIS A251, GLY A282	LEU A391
MO-10	−7.2	−55.82	GLU A523, THR A399	ILE A614, LYS A634, TYR A344, THR A398, LEU A525
MO-11	−7.2	−48.61	LYS A634, GLU A602	ILE A614, LYS A394, TYR A344, THR A399, ILE A397, LEU A525
MO-12	−7.2	−45.46	ALA A410	VAL A470, PHE A466, LEU A414

**FIGURE 8:**
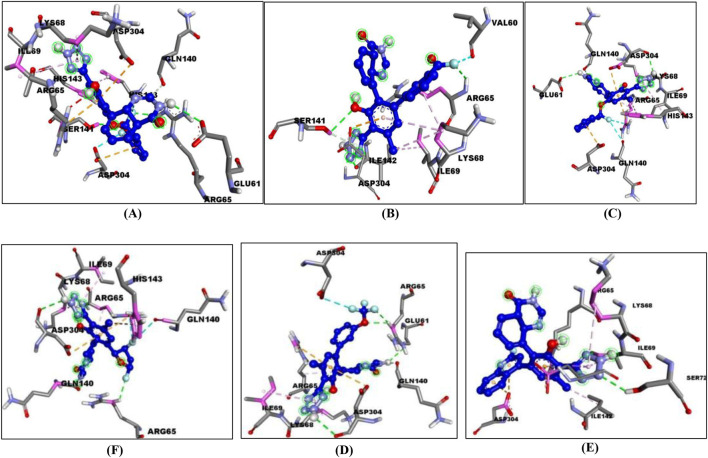
Three-dimensional (3D) interaction diagrams of methyl orsellinate analogues (MO-1 to MO-6) docked at the allosteric site of penicillin-binding protein 2a (PBP2a) from methicillin-resistant *Staphylococcus aureus* (MRSA). **(A)** MO-1 **(B)** MO-2 **(C)** MO-3. **(D)** MO-4 **(E)** MO-5 **(F)** MO-6.

**FIGURE 9:**
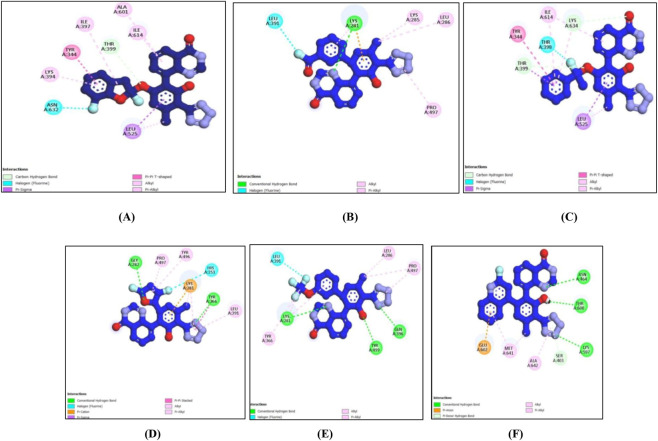
Two-dimensional (2D) interaction diagrams of methyl orsellinate analogues (MO-1 to MO-6) docked at the active site of penicillin-binding protein 2a (PBP2a) from methicillin-resistant *Staphylococcus aureus* (MRSA), highlighting major ligand–protein interactions. **(A)** MO-1 **(B)** MO-2 **(C)** MO-3. **(D)** MO-4 **(E)** MO-5 **(F)** MO-6.

**FIGURE 10:**
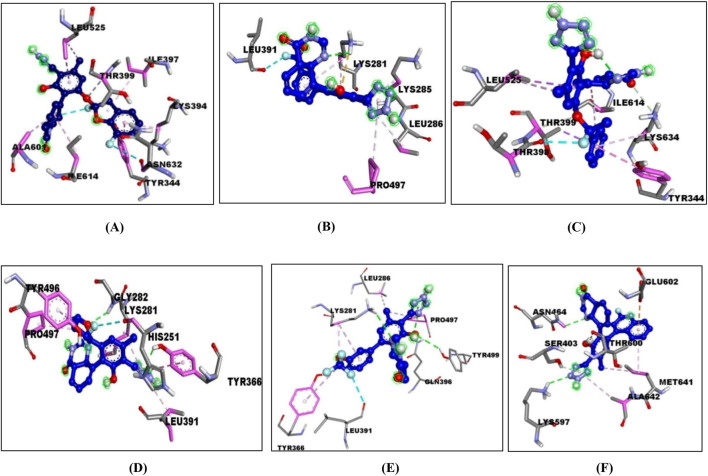
Three-dimensional (3D) interaction diagrams of methyl orsellinate analogues (MO-1 to MO-6) docked at the active site of penicillin-binding protein 2a (PBP2a) from methicillin-resistant *Staphylococcus aureus* (MRSA). **(A)** MO-1 **(B)** MO-2 **(C)** MO-3. **(D)** MO-4 **(E)** MO-5 **(F)** MO-6.

At the allosteric site, MO-2 exhibited a binding score of −9.1 kcal/mol. In the ADV docked complex, LYS A68 residue interacts at three different sites of the analogue; it forms three alkyl/π-alkyl interactions with the MO benzene ring, the methyl group of the MO ring and the fluorinated benzene ring, respectively (Figures 7, 8). ARG A65 forms an alkyl interaction with the fluorinated benzene ring and a hydrogen bond with its fluorine, while ILE69 forms an alkyl interaction with the methyl group of the MO benzene. ASP B304 interacts with the MO ring via π-anion interaction, and ILE A142 forms a hydrogen bond with the N atom of the tetrazole ring. SER B141 forms two interactions, a hydrogen bond with the O atom of the MO ring and a π-donor hydrogen bond with N of the tetrazole ring. Finally, VAL A60 forms a halogen bond with the fluorine atom. MO-2 demonstrated a binding score of −8.1 kcal/mol at the active site. In the docked complex of MO-2, LYS A281 forms three interactions with the analogue: a hydrogen bond with N atom of the isoquinolinone ring system, an alkyl interaction with the benzene ring and a π-cation interaction with the MO benzene ring (Figures 9, 10). Furthermore, both LYS A285 and LEU A286 form alkyl interactions with the methyl group at the C6 position of the MO ring. PRO A497 interacts with the imidazole ring of MO with an alkyl interaction. Finally, LEU A391 forms a halogen interaction with the fluorine atom (Tables 5, 6).

### 3.7 Molecular dynamics simulations

**FIGURE 11:**
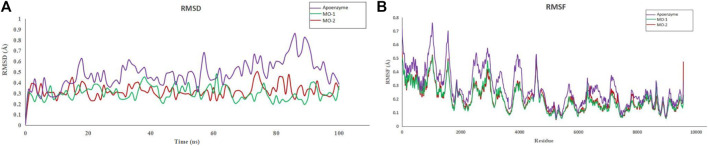
**(A)** Root mean square deviation (RMSD) and **(B)** root mean square fluctuation (RMSF) plots obtained from molecular dynamics (MD) simulations of MO-1 and MO-2 complexes in comparison with the unbound apoenzyme form of penicillin-binding protein 2a (PBP2a).

MD simulations were performed on the top two docked PBP2a-ligand complexes to assess dynamic stability and binding interactions using the CHARMM36 all-atom force field. Results revealed trajectories that were analysed for structural stability (RMSD, RMSF, radius of gyration), hydrogen bonding, and MM/PBSA binding free energies using Schrodinger-2024 Desmond software tools on high-performance computing clusters (Figure 11).

In the RMSD plot, both the complexes show quick equilibration after initial spikes (0–20 ns), with protein backbone plateauing at 0.2–0.4 Å. MO-2 shows slightly steadier ligand RMSD (lower fluctuations), while MO-1 maintains comparable overall compactness, indicating tightly folded, stable conformations throughout the simulation. The RMSF plot compares bound versus unbound states per residue. The RMSF values for MO-1 hover tightly between 0.1–0.5 Å for most residues, with subtle peaks up to 0.6 Å, confirming superior rigidity and compaction. Whereas the unbound apoenzyme shows slightly higher baseline (0.2–0.6 Å), and more variance, indicating overall looseness.

**FIGURE 12:**
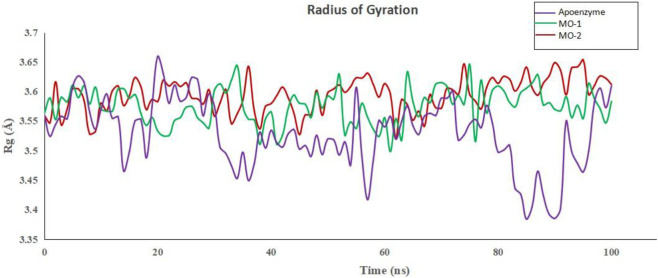
Radius of gyration (Rg) plots obtained from molecular dynamics (MD) simulations of MO-1 and MO-2 complexes compared with the unbound apoenzyme form of penicillin-binding protein 2a (PBP2a), indicating overall protein compactness and structural stability.

The radius of gyration analysis reflected the overall compactness of the protein structures. MO-1 maintained a consistently low Rg range (3.52–3.60 Å), indicating a tightly folded and stable conformation. MO-2 exhibited slightly higher variability (3.55–3.65 Å), reflecting transient structural loosening. The apoenzyme showed higher and more irregular Rg values, suggesting reduced compactness in the unbound state. Thus, MO-1 contributes to a more compact and stable global fold (Figure 12).

**FIGURE 13:**
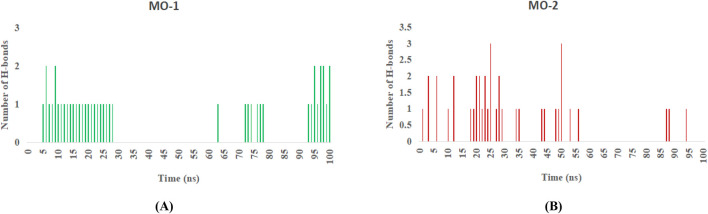
Number of hydrogen bonds formed during molecular dynamics (MD) simulations for **(A)** MO-1 and **(B)** MO-2 complexes with penicillin-binding protein 2a (PBP2a), indicating interaction stability throughout the simulation period.

**FIGURE 14:**
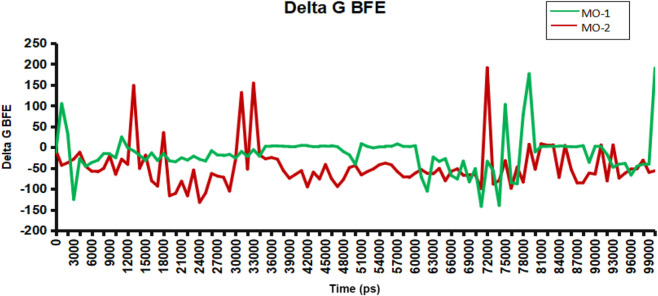
Gibbs free binding energy profiles of MO-1 and MO-2 complexes with penicillin-binding protein 2a (PBP2a) obtained during molecular dynamics (MD) simulations.

**FIGURE 15:**
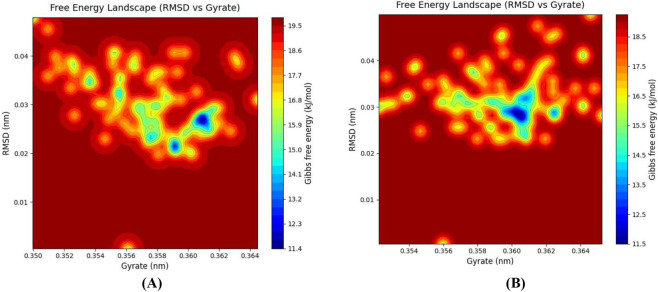
Free energy landscape (FEL) analysis for **(A)** MO-1 and **(B)** MO-2 complexes with penicillin-binding protein 2a (PBP2a), illustrating conformational stability and energetically favourable states during molecular dynamics (MD) simulation.

Hydrogen bonding, defined by donor–acceptor distance <3.5 Å and bond angle >120°, provides evidence of interaction persistence. Both ligands maintained intermittent yet consistent hydrogen bonds throughout the simulation. MO-1 exhibited 1–3 stable hydrogen bonds, particularly between 20–60 ns and 80–100 ns, whereas MO-2 showed more transient bonding, especially between 60 and 80 ns (Figure 13). The sustained interactions in MO-1 suggest improved stability and stronger protein–ligand affinity. Hydrogen bond timelines show intermittent interactions because of PBP2a’s dynamic nature and partially solvent-exposed pocket. Other interactions, including hydrophobic contacts, π–π stacking, and electrostatic interactions, compensate for them. These interactions all contribute to the complex’s stability, as shown by its favourable MM/PBSA binding energy, low RMSD, and consistent compactness. Binding thermodynamics was evaluated using MM/PBSA calculations, with free energy values ranging from −150 to +200 kJ/mol. For most part of the trajectory, MO-1 continuously showed negative values (−50 to −150 kJ/mol), indicating stable and spontaneous binding. For the MO-1–PBP2a complex, MM/PBSA analysis produced an average ΔGbinding of −47.33 kJ/mol, showing favorable binding driven by ΔEmm value of −123.36 kJ/mol resulting from Van der Waals and electrostatic interactions. Hydrophobic stabilization is supported by non-polar solvation (ΔGnonpolar = −11.26 kJ/mol), while polar solvation energy of +87.29 kJ/mol opposes this interaction as expected because of the desolvation penalty. Excellent binding affinity is shown by the substantial ΔEmm. MO-1 is positioned as a drug-like candidate for more research due to its reasonably significant ΔGbinding. Less favourable interactions were indicated by MO-2’s wider fluctuations and frequent crossings of zero. For MO-1, lower ΔG values and smaller fluctuations indicate better thermodynamic stability and binding affinity (Figure 14). MO-1 formed a single, deep energy basin with a minimum Gibbs free energy (∼11.4 kJ/mol) and a compact distribution (Rg = 0.358–0.360 nm, RMSD = 0.022–0.028 nm), indicating a stable conformation, according to free energy landscape (FEL) analysis using RMSD and Rg as reaction coordinates (Supplementary Figure S4 of Supplementary Material). MO-2, on the other hand, showed several shallow minima over wider RMSD (0.025–0.034 nm) and Rg (0.356–0.362 nm) ranges, suggesting more flexibility and less stable equilibrium states (Figure 15).

## 4 Discussion

This study combines phytochemical insights with sophisticated computational techniques to identify new inhibitors targeting PBP2a, the primary determinant of β-lactam resistance in MRSA. GC-MS analysis identified a total of 20 bioactive compounds in *P*. *perlatum*, with prominent peaks corresponding to MO and related phenolic derivatives, indicating their high abundance and significance. FTIR analysis revealed characteristic absorption peaks for hydroxyl (–OH), carbonyl (C=O), and C–O functional groups, confirming the presence of phenolics and flavonoids. Together, these findings indicate a phenolic-rich extract, supporting its potential antimicrobial activity. The results show that rational scaffold modification of MO greatly improves its pharmacokinetic appropriateness, binding affinity, and dynamic stability, confirming the usefulness of bioisosteric design in the search for antimicrobial drugs.

The higher performance of the analogue MO-1 across several computational parameters is one of the study’s main highlights. MO-1 showed much better binding affinities at both the active (−8.4 kcal/mol) and allosteric (−9.8 kcal/mol) sites of PBP2a when compared to the parent drug, which showed comparatively weak docking scores. The deliberate addition of heterocyclic and fluorinated moieties, which enhanced electronic distribution, steric complementarity, and interaction potential with crucial amino acid residues like LYS68, ASP304, THR399, and ILE614, is responsible for this improvement. A stable ligand-protein complex was produced by these interactions, which included hydrogen bonding, π–π stacking, and halogen bonding. This underscores the significance of specific structural changes in overcoming intrinsic resistance mechanisms. Importantly, the dual-site binding approach used in this investigation aligns with the known mechanism of action of CFT, a fifth-generation cephalosporin that exploits PBP2a’s allosteric activation. MO-1 appears to replicate this strategy by engaging both the catalytic and allosteric domains simultaneously, potentially inducing conformational changes that expose the otherwise blocked active site. MO-1’s total binding energy exceeds CFT’s, indicating a more robust and stable interaction profile. This finding supports the idea that dual-site targeting is a viable strategy to combat resistance, especially in proteins like PBP2a where structural dynamics are essential for regulation.

The docking results were further supported by MD simulations, which shed light on the conformational behaviour and temporal stability of the ligand-protein complexes. Throughout the 100 ns simulation, the MO-1–PBP2a complex showed low RMSD values (∼0.3 nm), indicating quick equilibration and persistent structural stability. Minimal residue-level changes were found by RMSF analysis, indicating that ligand binding plays a role in the overall stiffness and stabilization of proteins. The complex’s structural integrity was further supported by the consistently low radius of gyration measurements, which verified the complex’s compactness. Thermodynamic investigations using MM/PBSA calculations showed mostly negative binding free energy values (−50 to −150 kJ/mol), which are suggestive of energetically favourable and spontaneous interactions. The MO-1 complex’s conformational stability is further demonstrated by the existence of a distinct, deep free energy basin (∼11.4 kJ/mol) in the FEL, indicating that it is in an energetically favourable state with few structural aberrations. MO-2, on the other hand, showed more variations and several shallow minima, indicating a poorer level of stability and binding consistency.

The assessment of MO-1 as a computationally prioritized candidate for future medication is further enhanced by the ADMET profile. Good oral bioavailability and systemic distribution are suggested by its high intestine absorption, favorable permeability, and acceptable clearance rates. Furthermore, reduced penetration of the blood-brain barrier lessens the possibility of negative effects associated to the central nervous system. MO-1’s anticipated AMES toxicity, however, suggests a possible mutagenesis risk that should not be disregarded. This limitation highlights the need for additional structural improvement and experimental validation, such as minimum inhibitory concentration (MIC) studies against MRSA strains and *in vitro* mutagenicity tests.

Overall, this study shows that scaffolds produced from lichen can be potential leads in the search for antimicrobial drugs when they are carefully altered. A thorough pipeline for lead optimization is created by combining scaffold morphing, dual-site docking, ADMET profiling, and long-timescale MD simulations. MO-1 in particular stands out as a computationally prioritised option for additional development, providing a possible means of addressing the escalating worldwide challenge of AMR through innovative, mechanism-driven design.

## 5 Limitations

The study offers a thorough computational analysis for the identification of potential PBP2a inhibitors, although, several limitations must be acknowledged. Firstly, the results are solely derived from *in silico* techniques, such as molecular docking, ADMET analysis, and MD simulations, which fail to enclose the full complexity of biological systems. Hence, the predicted binding affinities and pharmacokinetic properties may not directly apply to *in vitro* or *in vivo* results, which needs to be further evaluated. Secondly, docking depends on a static protein structure, which makes it hard to show how flexible the structure can be. While MD simulation solves this problem to some extent, but the 100 ns timescale may not be sufficient enough to capture long-term dynamics or rare binding events. Third, ADMET predictions are based on models and may not be correct. As stated earlier, the predicted AMES toxicity of MO-1 needs to be tested in the lab. The lack of experimental validation, such as MIC and cytotoxicity assays, further diminishes confidence in antimicrobial efficacy and safety.

Finally, the study examines a constrained chemical space, comprising merely twelve analogues, limiting lead optimization. The results point to some computationally prioritised candidates, clinical potential yet need confirmation through *in vitro* experiments, more simulations, and broader chemical investigations.

## 6 Conclusion

This study evaluates the stability and binding of MO analogues (MO-1 and MO-2) against PBP2a in MRSA. Using molecular docking, ADMET profiling, and 100 ns MD simulations, distinct stability patterns and binding characteristics for the two ligands were identified. MO-1 demonstrated superior conformational stability, reflected by lower RMSD and RMSF values, a consistently compact radius of gyration, and more persistent hydrogen bonding interactions compared to MO-2. The MM/PBSA binding free energy profile of MO-1 further revealed predominantly negative ΔG values, suggesting spontaneous and energetically favourable binding throughout the simulation. FEL analysis showed a single deep basin for MO-1, confirming higher conformational stability than MO-2. Overall, MO-1 showed superior stability and binding energetics, supporting its potential as a lead scaffold for anti-MRSA drug design.

## Data Availability

The datasets presented in this study can be found in online repositories. The names of the repository/repositories and accession number(s) can be found in the article/Supplementary Material.

## References

[B1] Alqahtani T. Adhikari A. Silva E. D. (2021). Calcium antagonistic mechanisms. Molecules 26, 1–17. 10.3390/molecules26216348 PMC8588472 34770756

[B2] Azam S. S. Abbasi S. W. (2013). Molecular docking studies for the identification of novel melatoninergic inhibitors for acetylserotonin-O-methyltransferase using different docking routines. Theor. Biol. Med. Model. 10 (1), 63. 10.1186/1742-4682-10-63 24156411 PMC3819668

[B3] Chiang Y. C. Wong M. T. Y. Essex J. W. (2020). Molecular dynamics simulations of antibiotic ceftaroline at the allosteric site of penicillin-binding protein 2a (PBP2a). Israel J. Chem. 60 (7), 754–763. 10.1002/ijch.202000012

[B4] Devi K. V. Bhargav E. Swaruparani G. Jyothi M. V. (2021). Comparative evaluation of phytochemical constituents by GC-MS and antitubercular & antimicrobial potential of Ceiba pentandra and Parmotrema perlatum against resistant strains. J. Pharm. Res. Int. 33, 197–203. 10.9734/jpri/2021/v33i35a31889

[B5] Elabed S. Ali M. Hanafy S. Mostafa S. Mamdouh M. Meselhi A. (2025). Integrated experimental and computational analysis reveals amoxicillin binding dynamics to PBP1a in *Staphylococcus aureus* . Sci. Rep. 15 (1), 20284. 10.1038/s41598-025-07626-x 40562774 PMC12198363

[B6] Fernando M. D. M. Adhikari A. Senathilake N. H. K. S. Silva E. D. D. Nanayakkara C. M. Wijesundera R. L. C. (2019). *In silico* pharmacological analysis of a potent anti-hepatoma compound of mushroom origin and emerging role as an adjuvant drug lead. Food Nutr. Sci. 10 (11), 1313–1333. 10.4236/fns.2019.1011095

[B7] Fu Y. Zhao J. Chen Z. (2018). Insights into the molecular mechanisms of protein-ligand interactions by molecular docking and molecular dynamics simulation: a case of oligopeptide binding protein. Comput. Math. Methods Med. 2018, 3502514. 10.1155/2018/3502514 30627209 PMC6305025

[B8] Gupta A. K. Vaishnav Y. Jain S. K. Annadurai S. Kumar N. (2024). Exploring novel apalutamide analogues as potential therapeutics for prostate cancer: design, molecular docking investigations and molecular dynamics simulation. Front. Chem. 12 (August), 1–23. 10.3389/fchem.2024.1418975 39165335 PMC11333239

[B9] Halder S. K. Mim M. M. Alif M. M. H. Shathi J. F. Alam N. Shil A. (2022). Oxa-376 and Oxa-530 variants of β-lactamase: computational study uncovers potential therapeutic targets of Acinetobacter baumannii. RSC Adv. 12 (37), 24319–24338. 10.1039/d2ra02939a 36128545 PMC9412156

[B10] Jiao F. Bao Y. Li M. Zhang Y. Zhang F. Wang P. (2023). Unraveling the mechanism of ceftaroline-induced allosteric regulation in penicillin-binding protein 2a: insights for novel antibiotic development against methicillin-resistant *Staphylococcus aureus* . Antimicrob. Agents Chemother. 67 (12), e0089523. 10.1128/aac.00895-23 37971241 PMC10720500

[B11] Khattab M. Al-Karmalawy A. A. (2021). Revisiting activity of some nocodazole analogues as a potential anticancer drugs using molecular docking and DFT calculations. Front. Chem. 9, 628398. 10.3389/fchem.2021.628398 33842429 PMC8024586

[B12] Masumi M. Noormohammadi F. Kianisaba F. Nouri F. Taheri M. Taherkhani A. (2022). Methicillin-resistant staphylococcus aureus: docking-based virtual screening and molecular dynamics simulations to identify potential penicillin-binding protein 2a inhibitors from natural flavonoids. Int. J. Microbiol. 2022, 9130700. 10.1155/2022/9130700 35571353 PMC9095385

[B13] McGreig J. E. Uri H. Antczak M. Sternberg M. J. E. Michaelis M. Wass M. N. (2022). 3DLigandSite: structure-based prediction of protein-ligand binding sites. Nucleic Acids Res. 50 (W1), W13–W20. 10.1093/nar/gkac250 35412635 PMC9252821

[B14] Mirzadeh A. Kobakhidze G. Vuillemot R. Jonic S. Rouiller I. (2022). *In silico* prediction, characterization, docking studies and molecular dynamics simulation of human p97 in complex with p37 cofactor. BMC Mol. Cell Biol. 23 (1), 39. 10.1186/s12860-022-00437-2 36088301 PMC9464413

[B15] Nautiyal V. Dubey R. C. (2021). FT-IR and GC-MS analyses of potential bioactive compounds of cow urine and its antibacterial activity. Saudi J. Biol. Sci. 28 (4), 2432–2437. 10.1016/j.sjbs.2021.01.041 33935568 PMC8071964

[B16] Otero L. H. Rojas-Altuve A. Llarrull L. I. Carrasco-López C. Kumarasiri M. Lastochkin E. (2013). How allosteric control of *Staphylococcus aureus* penicillin binding protein 2a enables methicillin resistance and physiological function. Proc. Natl. Acad. Sci. U. S. A. 110 (42), 16808–16813. 10.1073/pnas.1300118110 24085846 PMC3800995

[B17] Pieroni M. Madeddu F. Di Martino J. Arcieri M. Parisi V. Bottoni P. (2023). MD–Ligand–Receptor: a high-performance computing tool for characterizing ligand–receptor binding interactions in molecular dynamics trajectories. Int. J. Mol. Sci. 24 (14), 11671. 10.3390/ijms241411671 37511429 PMC10380688

[B18] Pires D. E. V. Blundell T. L. Ascher D. B. (2015). pkCSM: predicting small-molecule pharmacokinetic and toxicity properties using graph-based signatures. J. Med. Chem. 58 (9), 4066–4072. 10.1021/acs.jmedchem.5b00104 25860834 PMC4434528

[B19] Pratibha P. Sharma Mahesh C. (2016). GC-MS analysis and biological activities of medicinally important lichen: parmelia perlata. Int. J. Pharmacogn. Phytochemical Res. 8 (12), 1975–1985.

[B20] Rahmadani A. Nindya Putri C. Khoirotul Wachidah A. Kholifah E. (2025). *In silico* evaluation of tamarind leaf flavonoids targeting ERα as anti-breast cancer agents using molecular docking. Indonesian J. Pharm. Educ. 5 (3), 343–356. 10.37311/ijpe.v5i3.33673

[B21] Sankaranarayanan P. G D. J. D. Pa A. Manikandan M. Ghosh A. (2024). Molecular docking and MD simulation approach to identify potential phytochemical lead molecule against triple negative breast cancer. F1000Research 13, 1271. 10.12688/f1000research.155657.1 40860264 PMC12375911

[B22] Thakur G. S. Gupta A. K. Pal D. Vaishnav Y. Kumar N. Annadurai S. (2025). Designing novel cabozantinib analogues as p-glycoprotein inhibitors to target cancer cell resistance using molecular docking study, ADMET screening, bioisosteric approach, and molecular dynamics simulations. Front. Chem. 13, 1543075. 10.3389/fchem.2025.1543075 40084274 PMC11903459

