# Supplementary material for: Pharmacokinetics, molecular docking, and molecular dynamics simulation unveil novel lichen-derived scaffolds targeting PBP2a MRSA
Source: Front Bioinform. 2026 Jun 10;6:1820903. doi: 10.3389/fbinf.2026.1820903 (PMC13291575; doi:10.3389/fbinf.2026.1820903)
Supplement: Supplementary file 1 [file Supplementaryfile1.docx]

***Supplementary Material***


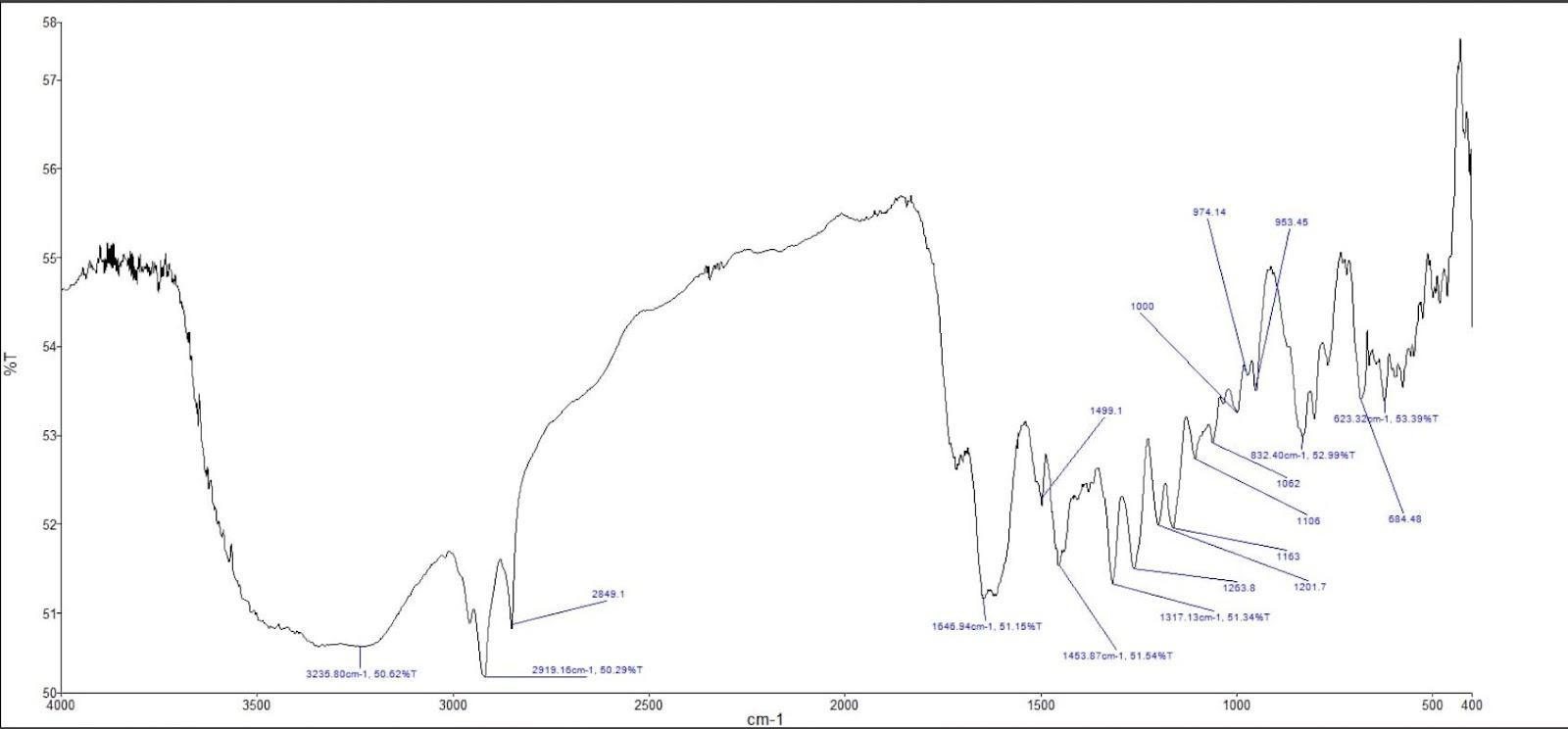


Figure S1: Infrared spectra of the bioactive fraction of the ethanolic extract of P. perlatum over a wave number ranging from 400 cm-1 to 4000 cm-1

**
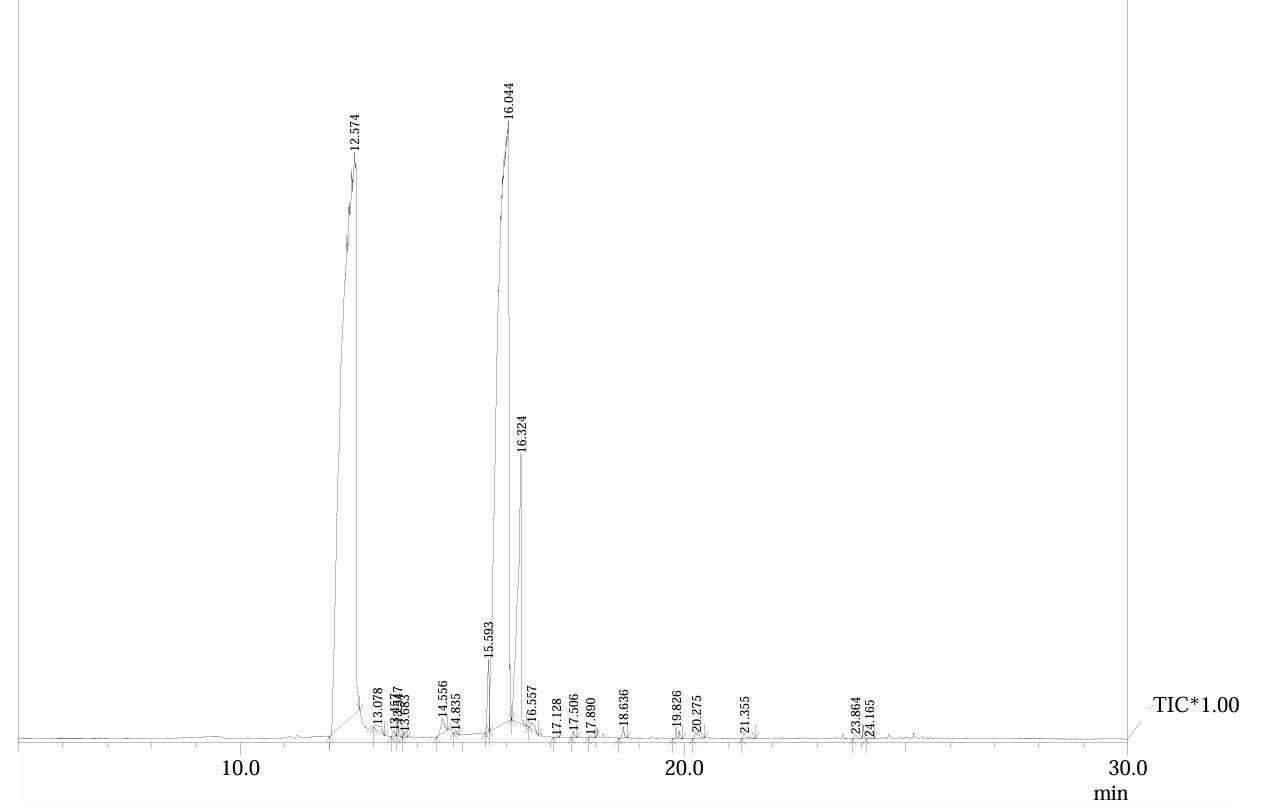
**

Figure S2: GC-MS results of the phytocompounds present in the lichen represented on a chromatogram.


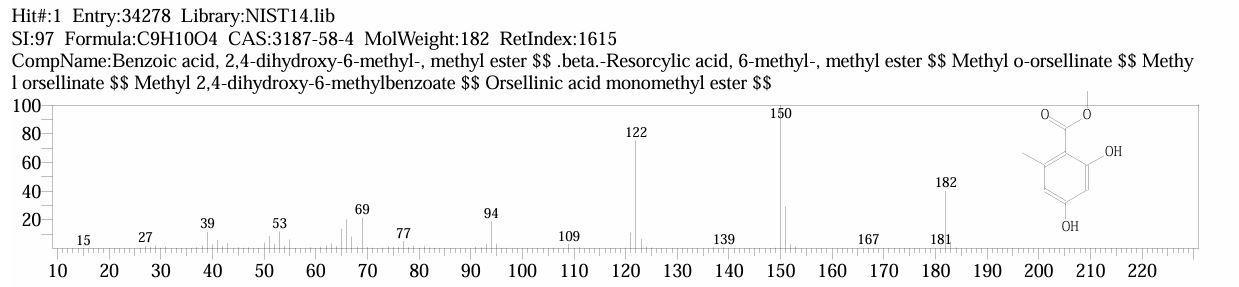


Figure S3: Peaks of Benzoic acid-2,4-dihydroxy-6-methyl methyl ester (MO)

**Table S1:** GC-MS results of the phytocompounds present in the lichen.

| **Peak** | **Retention**  **Time** | **Area** | **Area**  **Percentage** | **Name of the Compound** |
| --- | --- | --- | --- | --- |
| 1 | 12.574 | 506402073 | 49.62 | 5-Methyl-1,3-benzenediol |
| 2 | 13.078 | 2280839 | 0.22 | 2-Deoxypentopyranose |
| 3 | 13.457 | 702098 | 0.07 | DL-Proline-5-oxo-methyl ester |
| 4 | 13.547 | 839733 | 0.08 | 3-Methoxy-5-propylphenol |
| 5 | 13.683 | 398681 | 0.04 | 3-Chloro-2,6-dihydroxy-4-methyl benzaldehyde |
| 6 | 14.556 | 3082189 | 0.30 | 2,6-Dihydroxy-4-methyl-benzaldehyde |
| 7 | 14.835 | 404836 | 0.04 | 1,4-Anhydro-D-mannitol |
| 8 | 15.593 | 8097080 | 0.79 | Benzoic acid-3-formyl-2,4-dihydroxy-  6-methyl- methyl ester |
| 9 | 16.044 | 429893477 | 42.13 | Benzoic acid-2,4-dihydroxy-6-methyl-methyl ester |
| 10 | 16.324 | 59791902 | 5.86 | Benzoic acid-2,4-dihydroxy-3,6-dimethyl-  methyl ester |
| 11 | 16.557 | 1873379 | 0.18 | D-Arabinitol |
| 12 | 17.128 | 195910 | 0.02 | Benzoic acid-2,4-dihydroxy-3,5,6-trimethyl-methyl ester |
| 13 | 17.506 | 524395 | 0.05 | 7-Acetyl-6-ethyl-1,1,4,4-tetramethyl tetralin |
| 14 | 17.890 | 318710 | 0.03 | 2H-Benzocyclohepten-2-one |
| 15 | 18.636 | 1129418 | 0.11 | n-Hexadecanoic acid |
| 16 | 19.826 | 1332768 | 0.13 | 9,12-Octadecadienoic acid methyl ester |
| 17 | 20.275 | 1478439 | 0.14 | Linoelaidic acid |
| 18 | 21.355 | 989519 | 0.10 | 8,9-Dehydro-9-formyl-cycloisolongifolen |
| 19 | 23.864 | 589150 | 0.06 | 2-Hydroxy-3-[.beta.-iodo-.beta.-isopropyl vinyl] -1,4-naphthol |
| 20 | 24.165 | 188393 | 0.02 | Phosphinoline, 1,2,3,4-Tetrahydro-1-Phenyl |


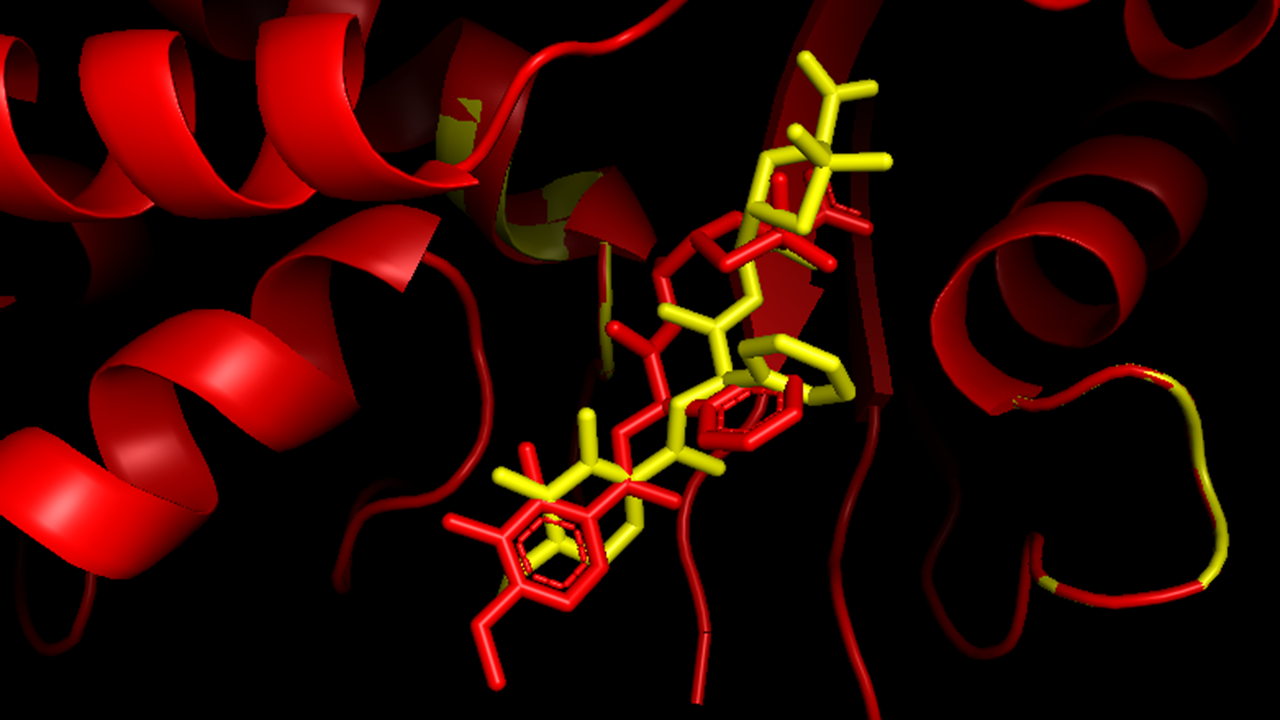

Figure S4: Superimposition of the co-crystallised (red) and re-docked (yellow) ligand within the active site of the target protein, visualised using PyMOL (RMSD= 0.00 Å)


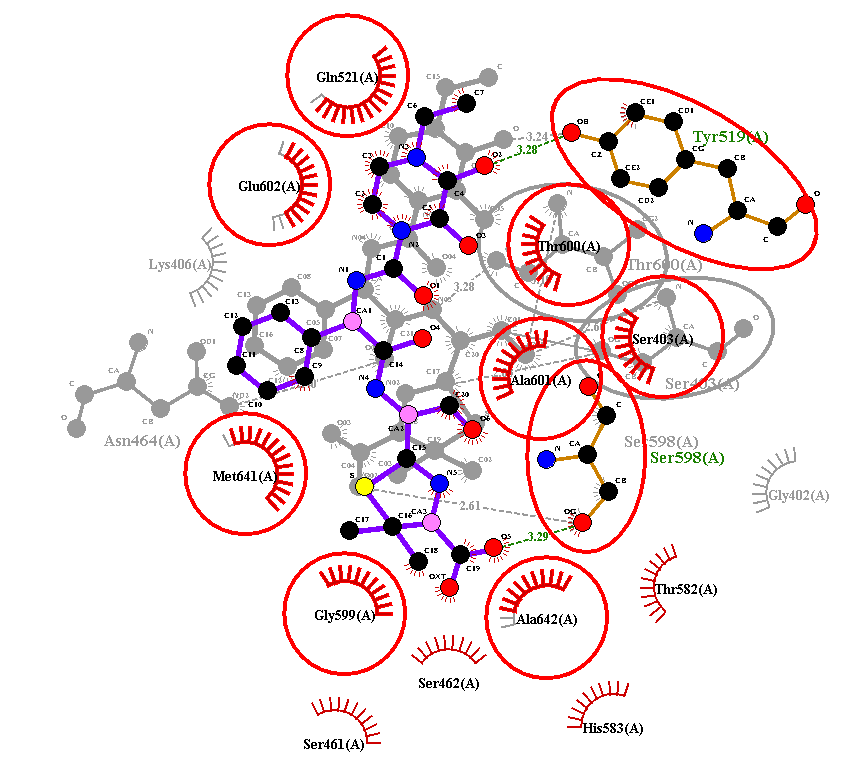


Figure S5: Re-docked ligand onto co-crystallized complex using LigPlot + v.2.2 showing superimposed amino acids (Red Circle)

# 7


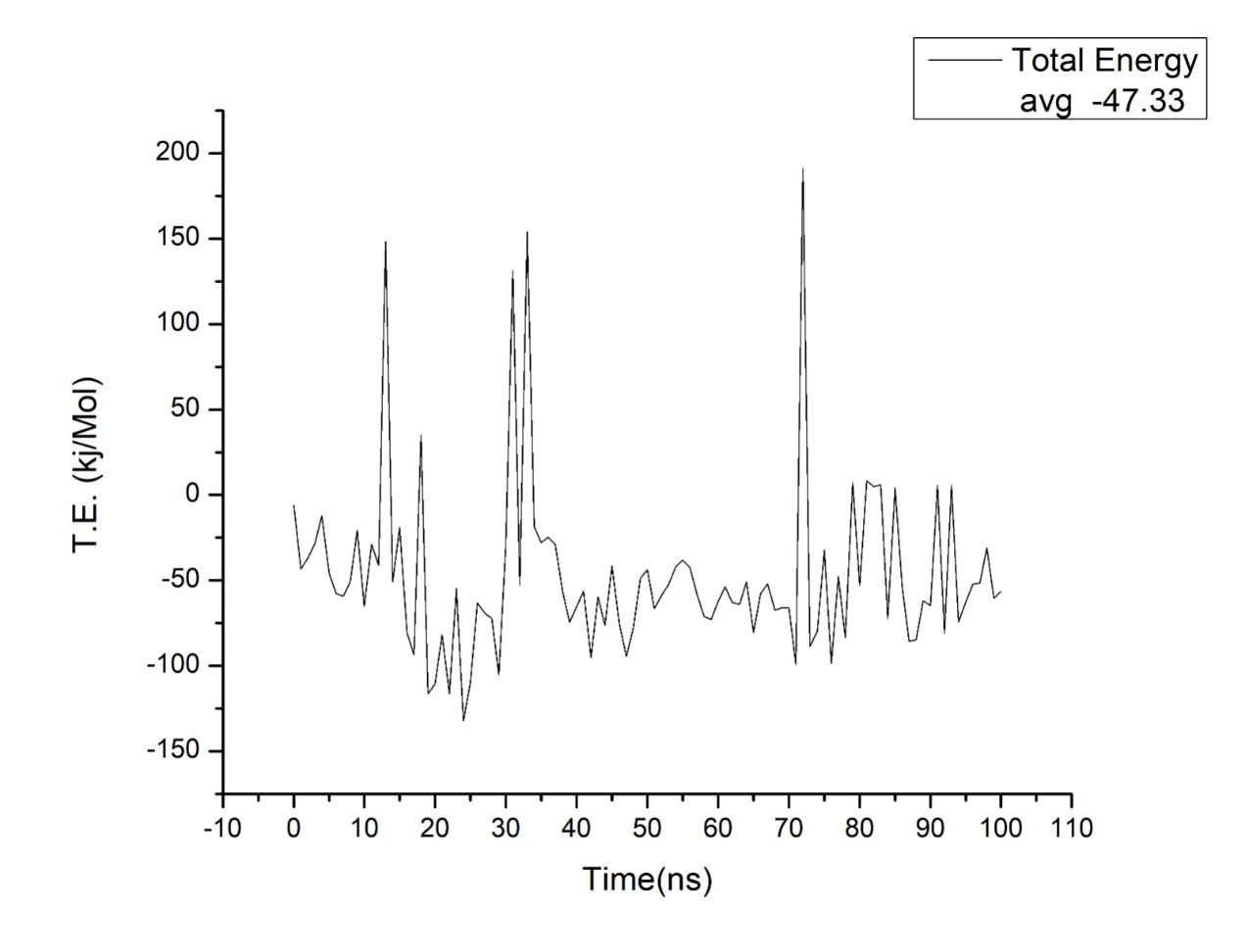


Figure S6: Total energy vs time graph
